# Radiation therapy at the end of life: a population-based study examining palliative treatment intensity

**DOI:** 10.1186/s13014-014-0305-4

**Published:** 2015-01-13

**Authors:** Marie-Adele Sorel Kress, Roxanne E Jensen, Huei-Ting Tsai, Tania Lobo, Andrew Satinsky, Arnold L Potosky

**Affiliations:** Huron River Radiation Oncology, 5301 E Huron River Dr Ann Arbor, Michigan, 48106 USA; Lombardi Comprehensive Cancer Center, Georgetown University Medical Center, 3300 Whitehaven St, NW Suite 4000, Washington, DC 20007 USA

**Keywords:** Palliative care, SEER-Medicare, Radiation therapy, Radiotherapy, Radiation oncology, End-of-life care

## Abstract

**Background:**

To examine factors associated with the use of radiation therapy (RT) at the end of life in patients with breast, prostate, or colorectal cancer.

**Methods:**

Using data from the Surveillance, Epidemiology, and End Results (SEER) – Medicare database, patients were over age 65 and diagnosed between January 1, 2004 and December 31, 2011 with any stage of cancer when the cause of death, as defined by SEER, was cancer; or with stage 4 cancer, who died of any cause. We employed multiple logistic regression models to identify patient and health systems factors associated with palliative radiation use.

**Results:**

50% of patients received RT in the last 6 months of life. RT was used less frequently in older patients and in non-Hispanic white patients. Similar patterns were observed in the last 14 days of life. Chemotherapy use in the last 6 months of life was strongly correlated with receiving RT in the last 6 months (OR 2.72, 95% CI: 2.59-2.88) and last 14 days of life (OR 1.55, 95% CI: 1.40-1.66). Patients receiving RT accrued more emergency department visits, radiographic exams and physician visits (all comparisons p < 0.0001).

**Conclusions:**

Among patients with breast, colorectal, and prostate cancer, palliative RT use was common. End-of-life RT correlated with end-of-life chemotherapy use, including in the last 14 days of life, when treatment may cause increased treatment burden without improved quality of life. Research is needed optimize the role and timing of RT in palliative care.

**Electronic supplementary material:**

The online version of this article (doi:10.1186/s13014-014-0305-4) contains supplementary material, which is available to authorized users.

## Background

In the last six months of life, cancer patients may undergo aggressive, costly care that often does not change their disease course, quality of life, or life expectancy [1,2]. Patients with breast, prostate and colorectal cancer often experience prolonged survival with metastatic or incurable disease, allowing time for a gradual shift in the focus of their medical care [3,4]. During this time, the goals of care can shift from cancer-directed treatment to comfort-directed care, addressing symptoms, physical function, and psychosocial health [5,6]. Treatment in this time period must achieve the dual aims of improving or sustaining quality of life, while minimizing treatment time and burden. Radiation therapy (RT) is commonly used to palliate symptoms of metastatic cancer or prevent impending severe morbidity, and single-fraction RT can be more cost effective than alternative methods of pain relief, including pain medication or chemotherapy [7-9]. However, RT requires daily treatment that can also cause limited short-term side effects, and it can require weeks after initiation of treatment to realize optimal palliation [10,11]. As a result, the timeliness of the use of RT is critical at the end of life: it needs to occur early enough to have meaningful clinical impact, and it may be inappropriate at the immediate-end of life due to high cost and burden of treatment time.

The American Society of Clinical Oncology (ASCO) added the use of chemotherapy in the last fourteen days of life as one of its Quality Oncology Practice Initiative (QOPI) measures, demonstrating the importance of limiting treatment burden toward the end of life [12,13]. Similarly, the American Society for Radiation Oncology (ASTRO) has released a guideline statement regarding the treatment of patients with bone metastases, a common site for treatment at the end of life [14]. However, no current RT guidelines exist regarding an appropriate paradigm for decision-making with respect to the utilization of RT at the end of life. Studies have demonstrated dependence of palliative RT use on multiple clinical and non-clinical factors [15-18]. However, these studies did not address the use of RT in the setting of patients with recurrent or progressive disease, nor did they specifically address treatment practices at the end of life, or the relationship between use of RT and other cancer-directed therapies, such as chemotherapy or surgery.

In this study, we address these issues in our examination of utilization of end-of-life RT among a population-based cohort of patients with breast, prostate, or colorectal cancer six months prior to death. We evaluated sociodemographic, clinical, health care environmental, and quality indicators associated with the use of RT during this time period. In particular, this study aimed to examine possible indicators of quality, such as treatment in the last 14 days of life.

## Methods

### Data source and study cohort definition

This study used data from the Surveillance, Epidemiology, and End Results (SEER) – Medicare linked database, which links persons in the SEER database with their clinical Medicare claims files. The SEER database is a database supported by the National Cancer Institute that includes information on patients diagnosed with cancer, about whom data was collected in 17 geographic areas covering 25% of the U.S. population [19]. The Medicare claims files include diagnostic and procedure codes for linked patients, and the linked database also provides sociodemographic information regarding persons in the database.

**Figure 1:**
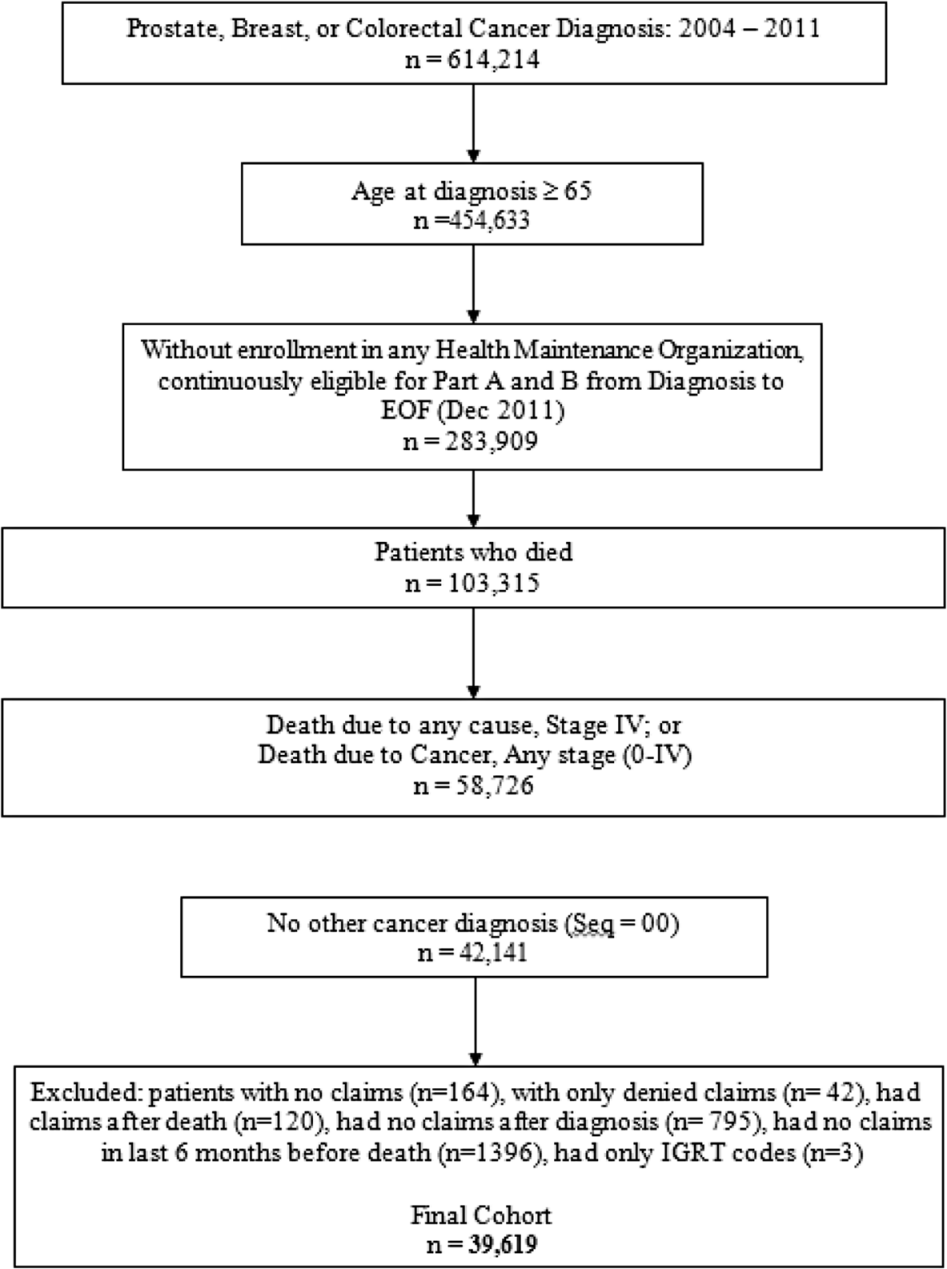
**Cohort development from SEER-Medicare linked database.**

We identified 614,214 patients diagnosed with prostate, breast, or colorectal cancer between January 1, 2004 and December 31, 2011 (Figure 1). Prostate, breast, and colorectal cancer were chosen for their high relative incidence and due to patients’ similar potential prolonged life with metastatic or advanced disease. With this group of three malignancies, the disease course and its subsequent treatment was more likely to have similar aims in the palliative setting. We investigated a cohort who either died with stage 4 cancer or died with cancer as the confirmed cause of death despite initial diagnosis of stage 0 – 3 disease between 2004 and 2011. Patients were included if they were diagnosed at age 65 or older (n = 454,633), were not treated in a Health Maintenance Organization and were continuously eligible for Part A and B Medicare (n = 283,909), and who died on or before December 31, 2011 (n = 103,315). Subjects were included if they died between January 1, 2004 and December 31, 2011 of any stage of cancer when the cause of death, as defined by SEER, was cancer; or who were initially diagnosed with stage 4 cancer and died of any cause during this time period (n = 58,726), and for whom the diagnosed cancer was the subject’s only malignancy or their first of no more than two malignancies (n = 42,141). We excluded subjects whom we suspected of errors in data linkage, or who had dual coverage (e.g., used VA services). Thus, we excluded cases who had only denied claims (n = 42), who had claims after death (n = 120), who had no claims after date of diagnosis (n = 795), who did not have any claims in the last six months before death (n = 1396). We excluded 5 cases that had only an Image Guided Radiation Therapy (IGRT) code due to possible cross-referencing with pure diagnostic, non-therapeutic imaging. The final cohort included 39,619 patients.

### Measures

Our primary outcome, palliative radiotherapy, was identified in patient claims files using Healthcare Common Procedure Coding System (HCPCS) codes (Additional file 1). Radiation therapy was considered in the last 6 months of life and separately in the last 14 days of life. Palliative radiotherapy was defined by a combination of patient cohort (all patients with stage 4 cancer would have been treated, by definition, with palliative intent) and cause of death (earlier-stage patients who died due to cancer).

Independent variables included the following clinical and sociodemographic factors: sex, race/ethnicity, age at diagnosis, marital status (unmarried, including: single, separated, divorced, or widowed; compared with married, including common law), Charlson index of comorbidity (calculated for two years prior to the last six months of life) [20]. We also included Census Tract level variables at the time of initial cancer diagnosis including: Region, Urban/Rural setting (divided into “Urban,” “Metro Urban,” and “Rural”), median household income (above/below median income of $43,000), and education (percent of persons over age 25 with a high school education only above/below median of 28%). Clinical variables included year of diagnosis, cancer type, stage at diagnosis (0–3, 4), cause of death, time from diagnosis until death and the use of prior radiation (radiation therapy received prior to the last 6 months of life). The use of surgery, chemotherapy, and radiologic examinations in the last 6 months were identified using a comprehensive list of HCPCS/Current Procedure Terminology (CPT) codes in the last 6 months of life (Additional file 1). Total inpatient days, emergency department visits, and physician visits were also calculated for the last six months of life.

Using a method developed by other investigators, patients were also designated as having had radiation in a freestanding facility versus a hospital-based or other type of facility by comparing the outpatient and carrier claims files [21].

### Statistical analyses

We first calculated summary statistics regarding the distributions of total inpatient days, emergency department visits, radiologic exams and physician visits to assess differences in utilization of healthcare between patients with and without palliative radiation use in the last 6 months of life. We then calculated percentages of palliative radiation use. Third, we performed chi-square tests for categorical variables and t-tests for continuous variable to compare the distribution of demographic and clinical factors between patients who did or did not get radiation in the last 6 months and 14 days of their lives. Fourth, we created multiple logistic regression models to identify those factors independently associated with palliative radiation use (as the binary dependent variable), adjusting for all other patients’ sociodemographics and clinical factors. Final results are presented as odds ratios with 95% confidence interval. All p-values were two-sided, and a p-value smaller than 0.01 was considered statistically significant. All analyses were conducted using the SAS 9.2 software (Cary, NC).

## Results

### Receipt of radiation in the last six months of life

Among the 39,619 patients included in this cohort, 50% received RT in the last six months of life. Bivariable analysis of RT use according to multiple sociodemographic, clinical, and health systems characteristics is presented in Table 1. Of patients aged 65–69, 63% received RT; rates of RT decreased through the oldest cohort, age > 85, with 38% receiving RT (trend from 65 to over 85, p < 0.0001). RT was used less commonly in non-Hispanic whites, as compared with all other racial/ethnic groups (p < 0.0001). Between 2004 and 2009, an increasing percentage of patients were treated with RT each year (p < 0.0001). When separated by stage, 54% of those with stage 0–3 cancer at diagnosis received RT in the last 6 months of life; 51% of those with stage 4 cancer received RT in the last 6 months of life (p < 0.0001). When evaluating the time alive from diagnosis until death, 55% of patients alive for 6 months or fewer received RT, while those alive for longer periods of time had rates of RT ranging from 46-48%.

**Table 1:** **Radiation therapy at the end of life: within six months or fourteen days of death**

	**All**	**6 Months**		**14 Days**	
	**n**	**n (%)**	**p value**	**n (%)**	**p value**
**Total**	39,619	19856 (50)		5723 (14)	
**Sex**	20,722		0.0183		<.0001
*Female*		10268 (50)		2834 (14)	
*Male*	18,897	9588 (51)		2889 (15)	
**Race/ethnicity**	30,904		<.0001		<.0001
*Non-Hispanic White*		15106 (49)		4279 (14)	
*Non-Hispanic Black*	4,791	2606 (54)		763 (16)	
*Hispanic*	2,250	1268 (56)		399 (18)	
*Other*	1,674	876 (52)		282 (17)	
**Race/ethnicity**	30,904		<.0001		<.0001
*Non-Hispanic White*		15106 (49)		4279 (14)	
*All others*	8,715	4750 (55)		1444 (17)	
**Age at diagnosis**	5,308		<.0001		<.0001
*Age 65 - 69*		3328 (63)		1071 (20)	
*Age 70 - 74*	6,154	3633 (59)		1065 (17)	
*Age 75 - 79*	7,367	4027 (55)		1160 (16)	
*Age 80 - 84*	8,599	4206 (49)		1138 (13)	
*85+*	12,191	4662 (38)		1289 (11)	
**Charlson index**	17,075		0.2403		0.0835
*0*		8628 (51)		2558 (15)	
*1*	10,203	5048 (49)		1436 (14)	
*2 or more*	12,341	6180 (50)		1729 (14)	
**Census region**	17,246		<.0001		<.0001
*West*		8768 (51)		2597 (15)	
*South*	15,733	7666 (49)		2129 (14)	
*North*	4,082	2111 (52)		633 (16)	
*East*	2,558	1311 (51)		364 (14)	
**Urban/rural**	20,695		<.0001		<.0001
*Urban*		10908 (53)		3312 (16)	
*Metro Urban*	11,506	5666 (49)		1575 (14)	
*Rural*	7,410	3279 (44)		835 (11)	
*Missing/unknown*	8	3 (38)		1 (13)	
**Median income**	19,689		0.0053		0.0107
*Above median (43 K)*		10017 (51)		2925 (15)	
*Below median (43 K)*	19,686	9739 (49)		2772 (14)	
*Missing/ unknown*	244	100 (41)		26 (11)	
**High school only**	19,693		<.0001		<.0001
*Above median (28%)*		9664 (49)		2707 (14)	
*Below median (28%)*	19,694	10096 (51)		2992 (15)	
*Missing/unknown*	232	96 (41)		24 (10)	
**Marital status**	21,554		<.0001		<.0001
*Unmarried*		10199 (47)		2796 (13)	
*Married*	15,354	8357 (54)		2553 (17)	
*Missing/unknown*	2,711	1300 (48)		374 (14)	
**Year of diagnosis**	8,903		<.0001		<.0001
*2004*		4257 (48)		1207 (14)	
*2005*	8,092	4009 (50)		1156 (14)	
*2006*	7,312	3611 (49)		996 (14)	
*2007*	6,239	3258 (52)		971 (16)	
*2008*	5,194	2674 (51)		790 (15)	
*2009*	3,879	2047 (53)		603 (16)	
**Cancer type**	8,629		<.0001		<.0001
*Breast*		4641 (54)		1292 (15)	
*Colorectal*	21,469	10318 (48)		3039 (14)	
*Prostate*	9,521	4897 (51)		1392 (15)	
**Stage**	11,115		<.0001		<.0001
*0-3*		5907 (53)		1634 (15)	
*4*	28,504	13949 (49)	<.0001	4089 (14)	<.0001
**Cause of death**	30,293	15740 (52)		4508 (15)	
*Cancer*					
*Non Cancer*	9,326	4116 (44)		1215 (13)	
**Time from diagnosis to death**	17,006		<.0001		<.0001
*0 days to 6 months*		9323 (55)		3089 (18)	
*6 months to 1 year*	6,065	2910 (48)		719 (12)	
*1 - 3 years*	12,160	5623 (46)		1407 (12)	
*More than 3 years*	4,388	2000 (46)		508 (12)	
**Prior radiation**	13,303		<.0001		<.0001
*No*		6157 (46)		2036 (15)	
*Yes*	26,316	13699 (52)		3687 (14)	
**Radiation Facility**	19,763				
*No radiation*		0 (0)		0 (0)	
*Hospital-based or Other*	14,442	14442 (100)		4330 (30)	
*Freestanding*	5,414	5414 (100)		1393 (26)	
**Surgery (last 6 months of life)**	25,873		<.0001		<.0001
*No*		12681 (49)		3616 (14)	
*Yes*	13,746	7175 (52)		2107 (15)	
**Chemotherapy**	1,585		<.0001		<.0001
*Last 14 days of life*		1169 (74)		577 (36)	
*Last 6 months of life, but not last 14 days*	10,999	7076 (64)		1655 (15)	
*None in last 6 months before death*	4,203	1694 (40)		396 (9)	
*Never*	22,832	9917 (43)		3095 (14)	

Fifty-two percent of patients with radiation prior to the last six months also had RT in the last 6 months of life, while only 46% of those with no prior history of RT received it at the end of life (p < 0.0001). Thirty-five percent of patients had surgery in the last six months of life, with proportionally more patients undergoing RT who also had surgery during the last 6 months of life (p < 0.0001).

Patients who received RT in the six months of life (n = 19,856) were compared with those not treated with RT during this time period (n = 19,763). There were significant differences in subjects’ utilization of additional health care resources, presented in Table 2. Mean total inpatient days in the last six months of life did not differ between the two groups. Emergency Department visits, radiologic exams, and physician visits were all higher among patients receiving RT at the end of life (p < 0.0001 for all three comparisons).

**Table 2:** **Health care resource utilization in the last six months of life**

	**All**	**No RT** ^**1**^	**Yes RT**	**p value**
**Total inpatient days**	25 (79)	22 (82)	27 (71)	0.38
*Median (standard deviation (SD))*				
**Total emergency department visits**	2 (3)	1 (3)	2 (3)	<.0001
*Median (SD)*				
**Total radiologic exams**	10 (11)	6 (7)	14 (11)	<.0001
*Median*				
**Total physician visits**	20 (21)	13 (15)	27 (23)	<.0001
*Median*				

^1^RT defined as radiation therapy within the last six months of life.

Our study included patients initially diagnosed with cancer stages 0–3 who died due to cancer and those initially diagnosed with stage 4 cancer who died of any cause. We initially explored these two groups using two separate multivariate logistic models with respect to the multiple factors related to the receipt of palliative RT at the end of life. We found that the association between sociodemographic and clinical factors and receipt of palliative RT was similar across the two groups (Additional files 2 and 3). Therefore, for subsequent analysis, we combined the groups and included stage at diagnosis as a covariate.

Multivariate analysis of the entire study cohort is presented in Table 3. Non-white patients were more likely to receive RT than non-Hispanic white patients (OR 1.18, CI 1.12-1.25). The odds of receiving RT decreased with increasing age, with patients over age 85 least likely to receive RT (OR 0.41, CI 0.38-0.44). RT was used more often in patients also receiving chemotherapy in the last 6 months of life (OR 2.78, CI 2.63-2.93) or surgery in the last 6 months of life (OR 1.17, CI 1.11-1.23). Patients with stage 4 were less likely to undergo RT than those initially diagnosed with stage 0–3 (OR 0.70, CI 0.66-0.73).

**Table 3:** **Multivariate model** ^1^ **: radiation in last six months of life** ^2^

	**OR**	**95% CI**		**P value**
**Race/ethnicity**				
*Non-Hispanic White*				
*All Others*	1.18	1.12	1.25	<.0001
**Age at diagnosis**				
*Age 65 - 69*				
*Age 70 - 74*	0.86	0.79	0.93	0.0002
*Age 75 - 79*	0.74	0.68	0.80	<.0001
*Age 80 - 84*	0.61	0.57	0.66	<.0001
*85+*	0.41	0.38	0.44	<.0001
**Charlson index**				
*0*				
*1*	1.01	0.96	1.07	0.6427
*2 or more*	1.07	1.01	1.13	0.0144
**Marital status**				
*Unmarried*				
*Married*	1.15	1.10	1.20	<.0001
**Cancer type**				
*Breast*				
*Colorectal*	0.65	0.62	0.69	<.0001
*Prostate*	0.82	0.76	0.88	<.0001
**Prior radiation**				
*No*				
*Yes*	1.45	1.37	1.53	<.0001
**Surgery (last 6 months of life)**				
*No*				
*Yes*	1.17	1.11	1.23	<.0001
**Chemotherapy (last 6 months of life)**				
*No*				
*Yes*	2.78	2.63	2.93	<.0001
**Stage at diagnosis**				
*0-3*				
*4*	0.70	0.66	0.73	<.0001

^1^Referent group is the first line in each category, unless otherwise indicated.

^2^Additional factors for which we controlled in the multivariate model included census region, urban/rural designation, education, and time alive from diagnosis until death.

### Receipt of radiation in the last fourteen days of life

5,723 patients (14%) received RT within the last fourteen days of life; sociodemographic and clinical factors related to their treatment are also presented in Table 1. Fourteen percent of non-Hispanic white patients received RT during this time period, while 17% of all other racial/ethnic groups received RT (p < 0.0001). Of patients age 65–69, 20% received RT in the last fourteen days of life; rates of RT decreased through the oldest cohort, age > 85, with 11% of patients in that age group receiving RT (p < 0.0001). Eighteen percent of patients alive for 6 months or fewer from diagnosis until death received RT, compared to 12% of all other groups (p < 0.0001). Among patients receiving chemotherapy in the last 14 days of life, 36% of these patients also received RT, while 9-15% of patients not receiving chemotherapy in the last 14 days received RT during this time period (p < 0.0001).

The results of multivariate analysis of radiation therapy use in the last fourteen days of life were overall similar to the findings for RT given during the last six months of life and are presented in Additional file 4. However, a few notable differences include that patients with prostate cancer approached the rate of radiation therapy use of breast cancer patients in the last fourteen days of life. Additionally, radiation therapy was less associated with other high-treatment-intensity interventions in the last fourteen days of life than in the last six months of life.

## Discussion

This study is the first to examine the use of RT at the end of life for major solid cancers with a relatively long natural history, including analysis of outcomes both for patients with metastatic cancer and those who died due to cancer after an initial diagnosis of non-metastatic disease. The goal of this study was to examine a generalizable group of patients who would be most likely to receive palliative, non-curative RT, regardless of stage at initial diagnosis. In our cohort of palliative patients, half of all patients received at least one course of RT within 6 months of death, and even in the last 14 days of life.

Radiation use decreased with increasing age and was less common among non-Hispanic white patients, after controlling for all other characteristics. These trends were consistent when looking both at the last six months and last fourteen days of life, and are similar to results reported in other studies of palliative radiotherapy [15-18], palliative chemotherapy [22], and end-of-life care in general [23,24]. One recent study examined the use of radiation therapy in the last 30 days of life among patients who died due to cancer and found significantly lower rates of radiation therapy use [16]. Our study demonstrated higher rates of utilization both due to the larger time window examined as well as the inclusion of patients dying from causes other than cancer. These differences in treatment practice according to age and racial/ethnic group cannot be attributed to relative life expectancy or comorbidity, and thus might represent unmeasured variables, such as patient or family preference or cultural beliefs. It is possible that older patients, as well as those who have been alive with cancer for a longer period of time, have lower RT utilization as part of a broader pattern of low resource utilization at the end of life.

RT use was also associated with higher utilization of health care resources, including chemotherapy use, surgery, physician visits, emergency department visits, and number of radiologic exams. Increased health care utilization at end of life has been shown to be an expensive, nuanced issue [1,2]. These findings have implications regarding the quality and appropriateness of care at the end of life. Well-timed palliative radiotherapy can alleviate cancer-related symptoms and thereby improve patients’ quality of life in a cost-effective manner. It is possible that this “window” of appropriate care includes the last 6 months of life, as examined in this study. It is impossible in this study to discern whether RT use prevented further interventions (i.e. additional surgery, chemotherapy, or hospitalizations), was part of a general pattern of “high-utilization” of health care resources at the end of life among selected patients, or affected patients’ quality of life. However, our data suggest that end of life RT conforms to similar utilization patterns as other cancer treatments, such as chemotherapy, either when comparing longer and shorter time windows (6 months vs. 14 days) or comparing patients diagnosed with metastatic vs. non-metastatic cancer. Cancer-directed treatment within the last 2 weeks of life may not always be appropriate. ASCO’s Quality Oncology Practice Initiative (QOPI) measures, for example, note that chemotherapy in the last fourteen days of life is considered low quality, possible detrimental end-of life care [12,13].

Clinically, tailoring end-of-life care remains a challenge related to anticipating date of death; measuring quality of life for both patients and caregivers; and communicating clear expectations among caregivers, patients, and patient families. Given the limited guidance about palliative RT for this patient group nearing end of life, future research is needed to explore and define appropriateness and value in end-of-life RT as part of a comprehensive end-of-life plan for patients with cancer.

The central limitation to our study relates to the definition of “palliative” radiation therapy using administrative claims data. Although the cohort was designed to include patients undergoing palliative radiotherapy, it is possible that some patients were treated with curative intent within 6 months of death. Since our findings persisted with sub-group analyses, including treatment within 14 days before death, and the magnitude of differences were similar between metastatic and non-metastatic patients, we are confident that this cohort was treated with palliative intent. Similarly, no information was available regarding physician decision-making, patients' presenting complaints, or patient preference in this data set. Additionally, patients with breast, prostate, or colorectal cancer frequently have extended survival even when they are deemed to have incurable disease, making even a large window of time (i.e. 6 months) before death reasonable for palliative care analysis. Hospice data was not included in this analysis, so our findings are only applicable to patients who were not enrolled in hospice in the intervals studied. The SEER-Medicare data set itself has limitations due to its use of claims-based data only for a population of older patients within a system that is entirely fee-for-service. Additionally, due to the nature of the SEER-Medicare data, we were unable to extract data regarding radiation dose nor determine the start and stop times of individual courses of RT.

## Conclusions

Among subjects with cancer diagnoses where a prolonged lifespan is common, even in the setting of incurable malignancies, the use of RT in the final six months of life was common. The use of RT appeared was associated with other cancer-directed treatments, including chemotherapy, at the end of life. Recently published guidelines highlight the importance of tracking the use of high-cost, high-treatment-intensity approaches at the end of life and carefully tailoring treatment plans to maximize quality of life and minimize treatment burden that does not provide value to quality of life [14,25]. More research is needed to refine clinical quality objectives for end of life cancer care, to optimize the role of RT as a palliative tool at the end of life, and to elucidate the psychosocial and unmeasured clinical factors influencing end-of-life care.

## Additional files

Additional file 1:
**Radiotherapy, surgical, chemotherapy, and radiology codes.**
Additional file 2:
**Radiation Therapy at the End of Life, by Stage at Diagnosis.**
Additional file 3:
**Multivariate Model** ^**1**^ **: Radiation in the Last 6 Months of Life, by Stage.**
Additional file 4:
**Multivariate Model** ^**1**^ **: Radiation in the Last Fourteen Days of Life.**
