# Supplementary material for: Radiation therapy at the end of life: a population-based study examining palliative treatment intensity
Source: Radiat Oncol. 2015 Jan 13;10:15. doi: 10.1186/s13014-014-0305-4 (PMC4314753; doi:10.1186/s13014-014-0305-4)
Supplement: Additional file 1: — Radiotherapy, surgical, chemotherapy, and radiology codes. [file 13014_2014_305_MOESM1_ESM.doc]

Additional file 1: Radiotherapy, surgical, chemotherapy, and radiology codes

Radiotherapy Codes

61793-61800, 63620-63621, 76355-77373, 77399, 77401-77499, 77520-77525, 77750-77799, 79400, 79420, 79440, 79445, 79900, 79005, 79101, 97403, 0037T, 0082T, 0083T, 0197T, G0173, G0242, G0243, G0251, G0256, G0261, G0338, G0339, G0340; ICD-9 procedure codes: 92.20 - 92.33, 92.39, 93.59; ICD-9 diagnosis codes: V58.0, V66.1, V67.1

Surgical HCPCS Codes

11600-11606, 11620-11624, 11626, 11640-11644, 11646, 11719-11721, 11730, 11732, 11740, 17260-17264, 17266, 17270-17274, 17276, 17280-17284, 19120, 19125, 19126, 19160, 19162, 19180, 19182, 19200, 19240, 19260, 19271, 19301-19307, 21920, 21925, 21930, 21935, 2290, 23075-23077, 24075-24077, 25076, 27047-27049, 27237-27360, 27365, 27615, 27618, 27619, 27645, 27647, 28043, 28045, 32480, 32482, 32484, 32500, 33120, 33130, 38570, 38571, 38700, 38720, 38724, 38740, 38745-38747, 38760, 38770, 32100, 32110, 32124, 32141, 44100, 44111, 44140, 44141, 44143-44147, 44150-44153, 44155-44158, 44160, 44200-44208, 44210-44213, 44188, 44365, 44366, 44369, 44393, 44394, 45100-45112, 45114, 45116, 45119, 45120, 45123, 45126, 45170, 45190, 45395, 47120, 47122, 47125, 47130, 47135, 47143, 47370, 47380, 47381, 47382, 49215, 52601, 52606, 52612, 52620, 52630, 52640, 55810, 55821, 55831, 55840, 55845, 55866, 55873, 61312, 61313, 61315, 61320, 61333, 61333, 61343, 61458, 61490, 61500, 61510, 61518, 61520, 61548, 61590, 63275, 63276, 63277, 63281, 63301-63303, 63308, 22305, 22310, 22315, 22325-22327, 22520-22525, 27125, 27130, 27132, 27193, 27218, 27220, 27222, 27226, 27227, 27230, 27232, 27235, 27236, 27238, 27240, 27244-27246, 27248, 27250, 27252, 27257, 27265, 27266, 27268, 27269, 17311-17315, 44005, 44120, 44121, 44125, 63011, 63015-63017, 63020, 63030, 63035, 63042, 63045-63048, 63055-63057, 63075, 63076, 63081, 63082, 63085, 63086, 63101, 63103

Chemotherapy codes

ICD-9 diagnosis codes: V58.0, E933.1, E930.7

ICD-9 procedure codes: 99.25

HCPCS codes: Q0083-Q0085, J7150, J8999, 964XX, 965XX, J9XXX, 0519F, 51720, 96400-96402, 96405, 96408*96417, 96420, 96422, 96423, 96425, 96440, 96445, 96542, 96545, 96549

Radiology codes

HCPCS codes: 70010-70559. 71010-71555, 72010-72295 (exclude: 72291, 72292), 73000-73725 (exclude: 74327, 74328, 74329, 74330, 74340, 74355, 74360, 74363), 74400-74485 (exclude: 74475, 74480, 74485), 74710-74775, 75552-75893 (exclude 75893), 76000-76499 (exclude 76010, 76098, 76140, 76376, 76377), 76506-76800, 76840-76886, 77051-77059, 77071-77084 (exclude 77071, 77076), 78000-78999 (exclude 78227)
