# Supplementary material for: Radiation therapy at the end of life: a population-based study examining palliative treatment intensity
Source: Radiat Oncol. 2015 Jan 13;10:15. doi: 10.1186/s13014-014-0305-4 (PMC4314753; doi:10.1186/s13014-014-0305-4)
Supplement: Additional file 2: — Radiation Therapy at the End of Life, by Stage at Diagnosis. [file 13014_2014_305_MOESM2_ESM.doc]

Additional file 2: Radiation Therapy at the End of Life, by Stage at Diagnosis.

|  | **All Stage 0-3** | **Radiation in last 6 months before death (Stages 0-3)** | | **All Stage 4** | **Radiation in last 6 months before death (Stage 4)** | |
| --- | --- | --- | --- | --- | --- | --- |
|  | **n** | **n (Row%)** | **p value** | **n** | **n (Row%)** | **p value** |
| **overall** |  |  |  |  |  |  |
| **totals** | 11,115 | 5907 (53) |  | 28,504 | 13949 (49) |  |
| **Sex** |  |  | 0.9576 |  |  | 0.0003 |
| *Female* | 6,722 | 3571 (53) |  | 14,000 | 6697 (48) |  |
| *Male* | 4,393 | 2336 (53) |  | 14,504 | 7252 (50) |  |
| **Race/Ethnicity** |  |  | 0.0002 |  |  | <.0001 |
| *Non-Hispanic White* | 8,838 | 4606 (52) |  | 22,066 | 10500 (48) |  |
| *Non-Hispanic Black* | 1,254 | 710 (57) |  | 3,537 | 1896 (54) |  |
| *Hispanic* | 595 | 351 (59) |  | 1,655 | 917 (55) |  |
| *Other* | 428 | 240 (56) |  | 1,246 | 636 (51) |  |
| **Race/Ethnicity** |  |  | <.0001 |  |  | <.0001 |
| *Non-Hispanic White* | 8,838 | 4606 (52) |  | 22,066 | 10500 (48) |  |
| *All others* | 2,277 | 1301 (57) |  | 6,438 | 3449 (54) |  |
| **Age at Diagnosis** |  |  | <.0001 |  |  | <.0001 |
| *Age 65 - 69* | 1,496 | 983 (66) |  | 3,812 | 2345 (62) |  |
| *Age 70 - 74* | 1,788 | 1086 (61) |  | 4,366 | 2547 (58) |  |
| *Age 75 - 79* | 2,267 | 1290 (57) |  | 5,100 | 2737 (54) |  |
| *Age 80 - 84* | 2,530 | 1275 (50) |  | 6,069 | 2931 (48) |  |
| *85+* | 3,034 | 1273 (42) |  | 9,157 | 3389 (37) |  |
| **Charlson Index** |  |  | 0.6951 |  |  | 0.0231 |
| *0* | 4,574 | 2412 (53) |  | 12,501 | 6216 (50) |  |
| *1* | 3,002 | 1613 (54) |  | 7,201 | 3435 (48) |  |
| *2 or more* | 3,539 | 1882 (53) |  | 8,802 | 4298 (49) |  |
| **Census Region** |  |  | 0.0043 |  |  | 0.0042 |
| *West* | 4,764 | 2606 (55) |  | 12,482 | 6162 (49) |  |
| *South* | 4,355 | 2233 (51) |  | 11,378 | 5433 (48) |  |
| *North* | 1,118 | 615 (55) |  | 2,964 | 1496 (50) |  |
| *East* | 878 | 453 (52) |  | 1,680 | 858 (51) |  |
| **Urban/Rural** |  |  | <.0001 |  |  | <.0001 |
| *Urban* | 5,741 | 3172 (55) |  | 14,954 | 7736 (52) |  |
| *Metro Urban* | 3,201 | 1678 (52) |  | 8,305 | 3988 (48) |  |
| *Rural* | 2,172 | 1056 (49) |  | 5,238 | 2223 (42) |  |
| *Missing/unknown* | 1 | 1 (100) |  | 7 | 2 (29) |  |
| **Median Income** |  |  | 0.5287 |  |  | 0.0034 |
| *Above median (43K)* | 5,513 | 2949 (53) |  | 14,176 | 7068 (50) |  |
| *Below median (43K)* | 5,579 | 2951 (53) |  | 14,107 | 6788 (48) |  |
| *Missing/ unknown* | 23 | 7 (30) |  | 221 | 93 (42) |  |
| **High School only** |  |  | 0.2662 |  | 6681 (48) | <.0001 |
| *Above median (28%)* | 5,665 | 2983 (53) |  | 14,028 |  |  |
| *Below median (28%)* | 5,431 | 2917 (54) |  | 14,263 | 7179 (50) |  |
| *Missing/ unknown* | 19 | 7 (37) |  | 213 | 89 (42) |  |
| **Marital status** |  |  | <.0001 |  |  | <.0001 |
| *Unmarried* | 6,194 | 3162 (51) |  | 15,360 | 7037 (46) |  |
| *Married* | 4,339 | 2461 (57) |  | 11,015 | 5896 (54) |  |
| *Missing/unknown* | 582 | 284 (49) |  | 2,129 | 1016 (48) |  |
| **Year of diagnosis** |  |  | <.0001 |  |  | <.0001 |
| *2004* | 3,036 | 1548 (51) |  | 5,867 | 2709 (46) |  |
| *2005* | 2,634 | 1391 (53) |  | 5,458 | 2618 (48) |  |
| *2006* | 2,230 | 1149 (52) |  | 5,082 | 2462 (48) |  |
| *2007* | 1,694 | 935 (55) |  | 4,545 | 2323 (51) |  |
| *2008* | 1,071 | 617 (58) |  | 4,123 | 2057 (50) |  |
| *2009* | 450 | 267 (59) |  | 3,429 | 1780 (52) |  |
| **Cancer Type** |  |  | <.0001 |  |  | 0.0013 |
| *Breast* | 2,688 | 1694 (63) |  | 5,941 | 2947 (50) |  |
| *Colorectal* | 6,989 | 3382 (48) |  | 14,480 | 6936 (48) |  |
| *Prostate* | 1,438 | 831 (58) |  | 8,083 | 4066 (50) |  |
| **Cause of death** |  |  |  |  |  | <.0001 |
| *Cancer* | 11,115 | 5907 (53) |  | 19,178 | 9833 (51) |  |
| *Non Cancer* | 0 | 0 |  | 9,326 | 4116 (44) |  |
| **Time from diagnosis to death** |  |  | <.0001 |  |  | <.0001 |
| *0 days to 6 months* | 3,198 | 1851 (58) |  | 13,808 | 7472 (54) |  |
| *6 months to 1 year* | 1,784 | 929 (52) |  | 4,281 | 1981 (46) |  |
| *1 - 3 years* | 4,481 | 2264 (51) |  | 7,679 | 3359 (44) |  |
| *Nore than 3 years* | 1,652 | 863 (52) |  | 2,736 | 1137 (42) |  |
| **Prior radiation** |  |  | <.0001 |  |  |  |
| *No* | 3,073 | 1426 (46) |  | 10,230 | 4731 (46) | <.0001 |
| *Yes* | 8,042 | 4481 (56) |  | 18,274 | 9218 (50) |  |
| **Radiation Facility** |  |  | <.0001 |  |  | <.0001 |
| *No radiation* |  |  |  | 14,555 |  |  |
| *Hospital-based or Other* | 4,340 | 4340 |  | 10,102 | 10102 |  |
| *Freestanding* | 1,567 | 1567 |  | 3,847 | 3847 |  |
| **Surgery (last 6 months of life)** |  |  | <.0001 |  |  | 0.0001 |
| *No* | 6,473 | 3338 (52) |  | 19,400 | 9343 (48) |  |
| *Yes* | 4,642 | 2569 (55) |  | 9,104 | 4606 (51) |  |
| **Chemotherapy** |  |  | <.0001 |  |  | <.0001 |
| *Last 14 days of life* | 480 | 367 (76) |  | 1,105 | 802 (73) |  |
| *Last 6 months of life, but not last 14 days* | 3,116 | 2125 (68) |  | 7,883 | 4951 (63) |  |
| *None in last 6 months before death* | 1,464 | 686 (47) |  | 2,739 | 1008 (37) |  |
| *Never* | 6,055 | 2729 (45) |  | 16,777 | 7188 (43) |  |
