# Supplementary material for: Radiation therapy at the end of life: a population-based study examining palliative treatment intensity
Source: Radiat Oncol. 2015 Jan 13;10:15. doi: 10.1186/s13014-014-0305-4 (PMC4314753; doi:10.1186/s13014-014-0305-4)
Supplement: Additional file 3: — Multivariate Model 1 : Radiation in the Last 6 Months of Life, by Stage. [file 13014_2014_305_MOESM3_ESM.doc]

Additional file 3: Multivariate Model^1^: Radiation in the Last 6 Months of Life, by Stage

|  | **Stages 0-3** | | | | **Stage 4** | | | |
| --- | --- | --- | --- | --- | --- | --- | --- | --- |
|  | **OR** | **95% CI** | | **P value** | **OR** | **95%  CI** | | **P value** |
|
| **Race/Ethnicity** |  |  |  |  |  |  |  |  |
| *Non-Hispanic White* |  |  |  |  |  |  |  |  |
| *All Others* | 1.13 | 1.02 | 1.26 | 0.0187 | 1.19 | 1.12 | 1.27 | <.0001 |
| **Age at Diagnosis** |  |  |  |  |  |  |  |  |
| *Age 65 - 69* |  |  |  |  |  |  |  |  |
| *Age 70 - 74* | 0.84 | 0.72 | 0.98 | 0.0224 | 0.87 | 0.79 | 0.95 | 0.0035 |
| *Age 75 - 79* | 0.76 | 0.66 | 0.88 | 0.0002 | 0.73 | 0.67 | 0.80 | <.0001 |
| *Age 80 - 84* | 0.61 | 0.53 | 0.71 | <.0001 | 0.61 | 0.56 | 0.67 | <.0001 |
| *85+* | 0.47 | 0.40 | 0.54 | <.0001 | 0.39 | 0.36 | 0.43 | <.0001 |
| **Charlson Index** |  |  |  |  |  |  |  |  |
| *0* |  |  |  |  |  |  |  |  |
| *1* | 1.07 | 0.97 | 1.18 | 0.194 | 0.99 | 0.93 | 1.06 | 0.7846 |
| *2 or more* | 1.07 | 0.97 | 1.18 | 0.1837 | 1.07 | 1.01 | 1.14 | 0.03 |
| **Census Region** |  |  |  |  |  |  |  |  |
| *West* |  |  |  |  |  |  |  |  |
| *South* | 0.95 | 0.87 | 1.05 | 0.3295 | 1.03 | 0.97 | 1.09 | 0.3905 |
| *North* | 0.99 | 0.85 | 1.14 | 0.8653 | 1.02 | 0.93 | 1.11 | 0.733 |
| *East* | 0.93 | 0.79 | 1.10 | 0.4125 | 1.13 | 1.00 | 1.26 | 0.0451 |
| **Urban/Rural** |  |  |  |  |  |  |  |  |
| *Urban* |  |  |  |  |  |  |  |  |
| *Metro Urban* | 0.89 | 0.81 | 0.98 | 0.0164 | 0.88 | 0.83 | 0.93 | <.0001 |
| *Rural* | 0.77 | 0.69 | 0.87 | <.0001 | 0.70 | 0.65 | 0.75 | <.0001 |
| **High School only** |  |  |  |  |  |  |  |  |
| *Above median (28%)* |  |  |  |  |  |  |  |  |
| *Below median (28%)* | 1.02 | 0.93 | 1.11 | 0.7493 | 1.04 | 0.98 | 1.11 | 0.1583 |
| **Marital status** |  |  |  |  |  |  |  |  |
| *Unmarried* |  |  |  |  |  |  |  |  |
| *Married* | 1.10 | 1.01 | 1.20 | 0.0297 | 1.17 | 1.10 | 1.23 | <.0001 |
| **Cancer Type** |  |  |  |  |  |  |  |  |
| *Breast* |  |  |  |  |  |  |  |  |
| *Colorectal* | 0.52 | 0.47 | 0.58 | <.0001 | 0.74 | 0.69 | 0.79 | <.0001 |
| *Prostate* | 0.70 | 0.61 | 0.82 | <.0001 | 0.90 | 0.83 | 0.97 | 0.0058 |
| **Prior radiation** |  |  |  |  |  |  |  |  |
| *No* |  |  |  |  |  |  |  |  |
| *Yes* | 1.52 | 1.37 | 1.69 | <.0001 | 1.41 | 1.33 | 1.50 | <.0001 |
| **Surgery (last 6 months of life)** |  |  |  |  |  |  |  |  |
| *No* |  |  |  |  |  |  |  |  |
| *Yes* | 1.21 | 1.10 | 1.34 | <.0001 | 1.18 | 1.12 | 1.25 | <.0001 |
| **Chemotherapy (last 6 months of life)** |  |  |  |  |  |  |  |  |
| *No* |  |  |  |  |  |  |  |  |
| *Yes* | 2.67 | 2.42 | 2.94 | <.0001 | 2.84 | 2.66 | 3.02 | <.0001 |
| **Time from diagnosis to death** |  |  |  |  |  |  |  |  |
| *0 days to 6 months* |  |  |  |  |  |  |  |  |
| *6 months to 1 year* | 0.52 | 0.45 | 0.60 | <.0001 | 0.43 | 0.39 | 0.46 | <.0001 |
| *1 - 3 years* | 0.40 | 0.35 | 0.45 | <.0001 | 0.32 | 0.30 | 0.34 | <.0001 |
| *Nore than 3 years* | 0.35 | 0.30 | 0.41 | <.0001 | 0.29 | 0.26 | 0.32 | <.0001 |

^1^ Referent group is first line in each category, unless otherwise indicated.
