# Supplementary material for: Radiation therapy at the end of life: a population-based study examining palliative treatment intensity
Source: Radiat Oncol. 2015 Jan 13;10:15. doi: 10.1186/s13014-014-0305-4 (PMC4314753; doi:10.1186/s13014-014-0305-4)
Supplement: Additional file 4: — Multivariate Model 1 : Radiation in the Last Fourteen Days of Life. [file 13014_2014_305_MOESM4_ESM.doc]

Additional file 4: Multivariate Model^1^: Radiation in the Last Fourteen Days of Life.

|  | **OR** | **95%  CI** | | **P value** |
| --- | --- | --- | --- | --- |
|
| **Race/Ethnicity** |  |  |  |  |
| *Non-Hispanic White* |  |  |  |  |
| *All Others* | 1.12 | 1.04 | 1.20 | 0.0019 |
| **Age at Diagnosis** |  |  |  |  |
| *Age 65 - 69* |  |  |  |  |
| *Age 70 - 74* | 0.81 | 0.73 | 0.89 | <.0001 |
| *Age 75 - 79* | 0.72 | 0.65 | 0.79 | <.0001 |
| *Age 80 - 84* | 0.59 | 0.53 | 0.65 | <.0001 |
| *85+* | 0.45 | 0.41 | 0.50 | <.0001 |
| **Charlson Index** |  |  |  |  |
| *0* |  |  |  |  |
| *1* | 1.01 | 0.93 | 1.08 | 0.8835 |
| *2 or more* | 1.02 | 0.95 | 1.10 | 0.6364 |
| **Census Region** |  |  |  |  |
| *West* |  |  |  |  |
| *South* | 0.97 | 0.90 | 1.04 | 0.3482 |
| *North* | 1.02 | 0.92 | 1.13 | 0.727 |
| *East* | 0.99 | 0.87 | 1.13 | 0.8585 |
| **Urban/Rural** |  |  |  |  |
| *Urban* |  |  |  |  |
| *Metro Urban* | 0.84 | 0.79 | 0.90 | <.0001 |
| *Rural* | 0.67 | 0.61 | 0.73 | <.0001 |
| **High School only** |  |  |  |  |
| *Above median (28%)* |  |  |  |  |
| *Below median (28%)* | 1.04 | 0.97 | 1.11 | 0.2748 |
| **Marital status** |  |  |  |  |
| *Unmarried* |  |  |  |  |
| *Married* | 1.27 | 1.19 | 1.35 | <.0001 |
| **Cancer Type** |  |  |  |  |
| *Breast* |  |  |  |  |
| *Colorectal* | 0.79 | 0.73 | 0.85 | <.0001 |
| *Prostate* | 0.92 | 0.84 | 1.01 | 0.0678 |
| **Prior radiation** |  |  |  |  |
| *No* |  |  |  |  |
| *Yes* | 1.10 | 1.02 | 1.18 | 0.0099 |
| **Surgery (last 6 months of life)** |  |  |  |  |
| *No* |  |  |  |  |
| *Yes* | 1.04 | 0.98 | 1.11 | 0.2154 |
| **Chemotherapy (last 6 months of life)** |  |  |  |  |
| *No* |  |  |  |  |
| *Yes* | 1.55 | 1.45 | 1.67 | <.0001 |
| **Time from diagnosis to death** |  |  |  |  |
| *0 days to 6 months* |  |  |  |  |
| *6 months to 1 year* | 0.45 | 0.41 | 0.49 | <.0001 |
| *1 - 3 years* | 0.39 | 0.36 | 0.43 | <.0001 |
| *Nore than 3 years* | 0.37 | 0.33 | 0.42 | <.0001 |
| **Stage at Diagnosis** |  |  |  |  |
| *0-3* |  |  |  |  |
| *4* | 0.818 | 0.764 | 0.877 | <.0001 |

^1^ Referent group is first line in each category, unless otherwise indicated.
